# Dynamics of stress generation and reduction in the nursing team at an oncology center

**DOI:** 10.1590/1518-8345.2874.3156

**Published:** 2019-07-18

**Authors:** Jorge Luiz Guedes Sant'ana, Mauricio Uriona Maldonado, Leila Amaral Gontijo

**Affiliations:** 1 Universidade Federal de Santa Catarina, Florianópolis, SC, Brasil.

**Keywords:** Occupational Stress, Nursing Personnel, Absenteeism, Quality of Health Care, Patient Safety, Mathematical Model, Estresse Ocupacional, Equipe de Enfermagem, Absenteísmo, Qualidade da Assistência à Saúde, Segurança do Paciente, Modelo Matemático, Estres Laboral, Equipo de Enfermeria, Ausentismo, Calidad de los Cuidados de Salud, Seguridad del Paciente, Modelo Matemático

## Abstract

**Objective:**

to represent the dynamics of stress generation, accumulation and reduction in the nursing team at an oncology therapy center.

**Method:**

a mathematical simulation model of system dynamics was developed based on data collection *in loco*. The model served to test the impact of three policies aimed at reducing stress in the team, namely i) increase in the service load; ii) increase in the size of shift teams and iii) reduction of service hours per bed.

**Results:**

the model showed that the policy of increasing the size of the team obtained the best results, with the absenteeism index stabilizing at 8%; staff at leave also stabilizing at 4-5 people per month, as well as accumulated stress reduced to baseline levels.

**Conclusion:**

measures to monitor physical and emotional demands, hiring staff, better technical training for so-called stressful activities, and a better distribution of tasks can be effective in reducing absenteeism rates and improving the quality of life of these workers.

## Abstract (pt)

**Objetivo:**

representar a dinâmica de geração, acumulação e dissipação do estresse na equipe de enfermagem em um centro de terapia oncológica.

**Método:**

um modelo de simulação matemático de dinâmica de sistemas foi desenvolvido com base na coleta de dados *in loco*. O modelo serviu para testar o impacto de três políticas direcionadas a reduzir o estresse na equipe: i) incremento na carga de atendimento; ii) incremento do tamanho das equipes de turno e iii) redução de horas de atendimento por leito.

**Resultados:**

o modelo demonstrou que a política de incremento de tamanho da equipe obteve os melhores resultados, sintetizados com o índice de absenteísmo, estabilizando-se em 8%; pessoal afastado também se estabilizando em 4-5 pessoas por mês, bem como o estresse acumulado reduzido aos níveis do cenário base.

**Conclusão:**

as medidas no sentido de acompanhamento das demandas físicas e emocionais, a contratação de pessoal, um melhor treinamento técnico para as atividades ditas estressantes e uma melhor distribuição de tarefas podem ser eficazes na redução dos índices de absenteísmo e melhorar a qualidade de vida desses trabalhadores.

## Abstract (es)

**Objetivo:**

representar la dinámica de generación, acumulación y disipación del estrés en el equipo de enfermería en un centro de terapia oncológica.

**Método:**

un modelo de simulación matemático de dinámica de sistemas fue desarrollado basado en la recolección de datos *in loco*. El modelo sirvió para testar el impacto de tres políticas dirigidas a reducir el estrés en el equipo: i) incremento en la carga de atendimiento; ii) incremento del tamaño de los equipos de turno y iii) reducción de horas de atendimiento por lecho.

**Resultados:**

el modelo demostró que la política de incremento de tamaño del equipo obtuvo los mejores resultados, sintetizados con el índice de ausentismo estabilizándose en 8%; personal alejado también estabilizándose en 4-5 personas por mes, así como el estrés acumulado reducido a los niveles del escenario base.

**Conclusión:**

las medidas en el sentido de acompañamiento de las demandas físicas y emocionales, la contratación de personal, mejor entrenamiento técnico para actividades llamadas estresantes y una mejor distribución de tareas pueden ser eficaces en la reducción de los índices de ausentismo y mejorar la calidad de vida de esos trabajadores.

## Introduction

Health professionals have faced significant changes in work organization and work relationships, having to cope with stress to meet the demands of modern professional life^(^ ^1^ ^)^. Work-related stress^(^ ^2^ ^)^ occurs when the worker reacts to demands and pressures that do not match their skills and knowledge and that challenge their ability to cope with stressful situations.

There are several effects of prolonged stress, such as depression and absenteeism, which in addition to representing physical and mental fatigue are signs of an aggravation to physical and emotional health^(^ ^1^ ^–^ ^3^ ^)^. In the hospital setting, the impact of occupational stress^(^ ^4^ ^)^ in the performance of nursing professionals has been an important concern for managers because, in addition to affecting physical and mental health, it also affects their performance.

Due to the stress and the consequences that it can generate in the workplace, managers need to find ways to reduce it in teams and thereby improve the quality of care through reducing absenteeism^(^ ^5^ ^)^. However, little is known about which managerial policies are more efficient and about the time needed to effectively reduce absenteeism caused by stress.

Thus, researchers of this study chose to apply a methodology derived from systemic thinking, which focuses on the analysis of problems in which technical, human, and organizational components interact through computer simulation known as system dynamics^(^ ^6^ ^)^. System dynamics can help in the explicit representation of the interrelationship between stressors and their effect on absenteeism. It can also point the way to better policies aimed at reducing the physical and emotional load of the nursing staff.

The purpose of this article is to represent the dynamics of stress generation, accumulation and reduction in the nursing team at an oncology therapy center. For this purpose, a mathematical model is used, following the methodology of the system dynamics.

The choice of this group of workers for the development of this model was due to the heavy physical, mental and emotional workload to which they are exposed, reflecting a sample strongly susceptible to illness at work. Sleep problems^(^ ^5^ ^)^, high levels of stress and exhaustion, and low job satisfaction are also common among nurses. Physical incapacity is not often the main reason for work leave, but it is presented as a preventive measure. Thus, the main contribution of this article is to provide a better understanding of the effectiveness of managerial policies aimed at reducing stress in nursing teams from the use of the model developed as a cost-effective test bench to evaluate the effectiveness of programs to improve quality of work or training sessions prior to implementation.

## Method

The simulation model was developed following the methodological procedure of the system dynamics^(^ ^6^ ^)^. System dynamics has been widely used to simulate problems in the areas of business and environmental management and energy planning, among others and more recently in several health problems^(^ ^7^ ^–^ ^11^ ^)^.

The system dynamics methodology uses differential equations to represent the behavior of the variables of interest in the model^(^ ^6^ ^)^, in which the main element is the so-called ‘stocks’. Stocks are the variables that define the state of the system and change their value by the influence of rates. For example, the number of workers (stock) increases as more workers are hired (entry rate) and decreases as workers are cut off (exit rate). Mathematically, a system dynamics model can be represented by equations of the type^(^ ^6^ ^)^:

(1) $$\frac{d}{dt}X=f(X,p)$$

Where:

*X* = vector of ‘n’ stocks; *dX/dt* = the net change rate of the vector *X; f* = n-dimensional and usually non-linear function; and *p* = parameter vector

The model represents a group of nursing professionals who work in an oncology center at a public hospital, consisting of six nurses and twenty one nursing technicians. Their shifts are 12 hours of work with 60 hours of rest. Each team consists of one nurse and four technicians. In order for the shifts to be complete, some professionals are obliged to reduce the rest period by performing up to 15 shifts, when the ideal number would be ten shifts per month.

The research was possible due to the collaboration of a group of twenty-seven nurses and nursing techniques from the oncology center, who helped in the construction of the model. Six “key informants” were chosen because of their exposure to the personal, psychosocial, and organizational conditions of the team. Each one is a member of service teams and their years of experience in the service range from 4 to 25.

To participate, all informants should have experience in nursing, work, and personal experiences that could represent the *status quo* of the team. First, all participants answered the validated Burnout Inventory questionnaire for Brazilian culture^(^ ^12^ ^)^, which assesses workload and stress and the result revealed high rates.

Thus, the six informants participated in three sessions, providing data and insights for constructing the model, each session lasting 60 to 120 minutes. One of the authors of this article facilitated discussions by elucidating the structure and process of stress generation, accumulation, and reduction at the study site, while translating the conversations into visual mapping of the model.

Some of the aspects discussed in the three sessions were: i) work routine; ii) complications that generated stress; iii) how to deal with the death of children (patients at the cancer center), loss and grief *per se* and iv) organizational issues such as overwork, lack of staff, and lack of fellowship.

Finally, after several critical discussions, a final structure of the model was generated and inserted in Stella Architect software (www.iseesystems.com).

The next step was to parameterize the model. For this, data were extracted from a research in the oncology center with nurses and nursing assistants. Based on these data, the model was adjusted to represent the baseline scenario.

The behavior of the baseline scenario was compared with the current situation of the oncology unit, and the ability of the model to reproduce the behavior observed by the team as well as the usual number of nurses on leave (approximately three) was verified. Finally, the model was used to test three policies for stress mitigation, according to previous research experiences^(^ ^13^ ^)^: i) increase in the service load (that is, in the number of beds served per shift); ii) increase the size of shift teams and iii) reduction of service hours per bed.

The research was approved by the Ethics Research Committee under number 80845417.3.0000.0121, of July 5, 2018 and was conducted according to the guidelines for research with humans of the National Health Council and Code of Ethics and Research. All subjects agreed to participate in the study, were informed about the purpose and origin of the study and signed an Informed Consent Form (ICF), according to Resolution 466/2012. The analysis and specification of the risks are contained in the project and in the said ICF and the researchers recognize the risks of the research and commit to reimburse any damages caused.

## Results

**Figure 1:**
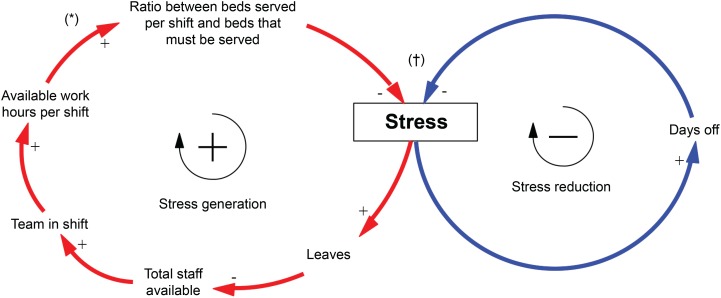
Diagram of causal loops. *The (+) symbol at the end of the arrow represents that the variables change in the same direction; †The (-) symbol at the end of the arrow represents that the variables change in the opposite direction

Figure 1 represents the simplified structure of the model, portraying the accumulation of stress (rectangle) as the result of two feedback loops, one of increasing effect, the one of stress generation (red loop) and the other one of decreasing effect, of stress reduction (blue loop).

Next, mathematical equations were formulated which, according to Figure 1, have an effect on accumulated stress. The formulated equations (to be presented in the Results) are the product of simple algebraic operations such as addition, subtraction, division and product. In other words, equations 2 to 10 were deduced by the authors for the explanation of the phenomena observed in them.

Thus, the accumulated stress is the difference between the stress generated in period t, $\gamma_{t}^{e}$ and the stress dissipated in the same period $\delta_{t}^{e}$, represented in the language of system dynamics by the following equation:

(2) $$\frac{d}{dt}\varepsilon_{t}=\gamma_{t}^{e}-\delta_{t}^{e}$$

The stress generation $\gamma_{t}^{e}$ is defined as the ratio between the number of beds to be served $\beta_{t}^{e}$ (the optimal rate of care performed scaled with teams of five nurses per shift) and the number of beds served per shift $\alpha_{t}^{e}$ (i.e., the actual rate of care performed):

(3) $$\gamma_{t}^{e}=\frac{\beta_{t}^{e}}{\alpha_{t}^{e}}$$

In other words, when the actual care capacity $\alpha_{t}^{e}$ is less than the scaled capacity $\beta_{t}^{e}$, the stress would accumulate above what it would have if the actual capacity met the demand completely.

The stress reduction $\delta_{t}^{e}$ responds proportionally to the accumulated stress *ε_t_* and inversely to the necessary shifts to dissipate stress, the constant *τ*, according to previous research^(^ ^13^ ^)^:

(4) $$\delta_{t}^{e}=\frac{\varepsilon_{t}}{\tau }$$

The accumulated stress *ε_t_* contributes to the increase in the absenteeism rate – in addition to other factors κ_1_ – redreducing the total available team $\rho_{t}^{d}$, and therefore compromising the formation of teams in shifts $\rho_{t}^{p}$, as shown by the following equations, where κ_1_, κ_2_, κ_3_ are constants:

(5) $$\phi_{t}^{a}=\varepsilon_{t}+\kappa_{1}$$

(6) $$\frac{d}{dt}\rho_{t}^{d}=\sigma_{t}^{d}-\phi_{t}^{a}$$

(7) $$\frac{d}{dt}\rho_{t}^{p}=\left(\rho_{t}^{d}\times \kappa_{2}\right)-\left(\rho_{t}^{p}\times \kappa_{3}\right)$$

The constraint on the formation of shift teams, in turn, reduces the available working hours per shift $h_{t}^{d}$ and this, in turn, reduced the number of beds served per shift $\alpha_{t}^{e}$, where κ_4_ is a constant and $h_{t}^{l}$ is a nonlinear function of accumulated stress :

(8) $$h_{t}^{d}=\rho_{t}^{p}\times \kappa_{4}$$

(9) $$\alpha_{t}^{e}=\frac{h_{t}^{d}}{h_{t}^{l}}$$

(10) $$h_{t}^{l}=f(\varepsilon_{t})$$

In summary, Figure 1 shows that when the reduction mechanism works properly, the system remains balanced. However, when the reduction mechanism is not fully effective or when stress is generated at a higher rate, the accumulated stress tends to grow, leading to more work leaves and, therefore, increasing the rate of absenteeism and illness in the team.

**Figure 2:**
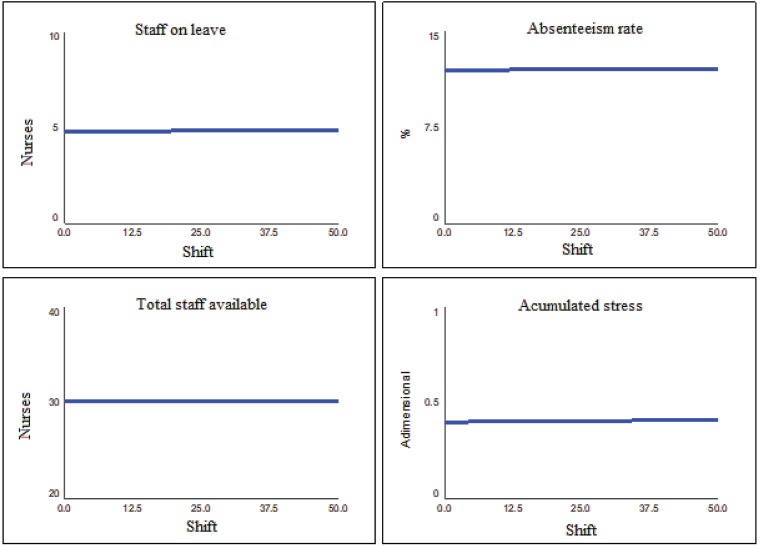
Baseline scenario reproducing the current behavior of the oncology unit

Figure 2 shows the result of the baseline scenario. The baseline scenario estimates an absenteeism rate of approximately 12%, five nurses on leave, 30 nurses in the total available staff, and an accumulated stress rate of 0.4 over the 50-shift period. In the baseline scenario, stress is not dissipated, remaining at a value other than zero, that is, representing a constant stress load. Also, the 12% absenteeism index is well above the 4% reference value identified in the literature, which means that at this level of stress the team will tend to suffer more in the long term than the average of the health sector.

**Figure 3:**
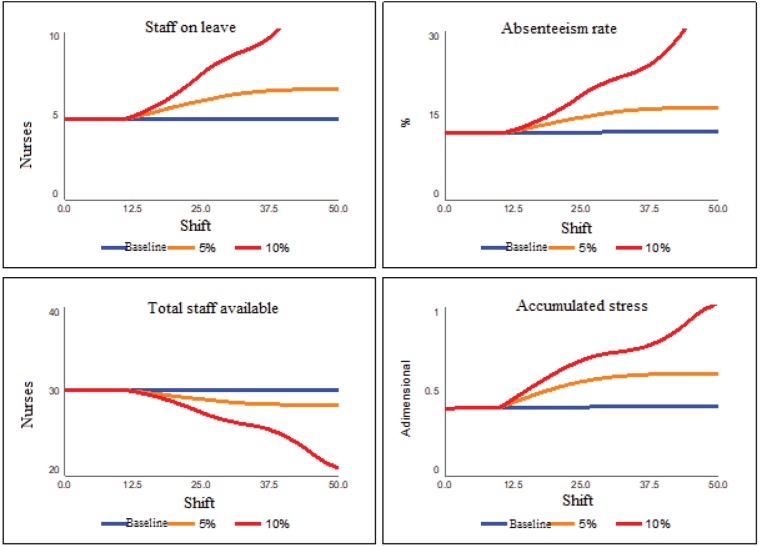
Increased workload scenarios

Once the model was explained and the baseline scenario was presented, some scenarios were simulated in order to verify the impact on the team's stress and absenteeism. The first set of scenarios establishes increases in the number of care performed by the oncology unit, that is, the effect of an increase in care performed is simulated, considering that the team remains fixed to five nurses per shift. The increments are 5% and 10%, regarding the number of beds that need care and start on shift 10. Figure 3 presents the results.

The results of Figure 3 show that, as shifts occur and there is an increase in workload, there is an increase in the number of staff on leave and in absenteeism, resulting in decreased teams and accumulated stress. This collapse behavior is more evident in the scenario of a 10% increase in the rate of care perfomed, leading to a 30% absenteeism index and a similar reduction of the total available staff.

**Figure 4:**
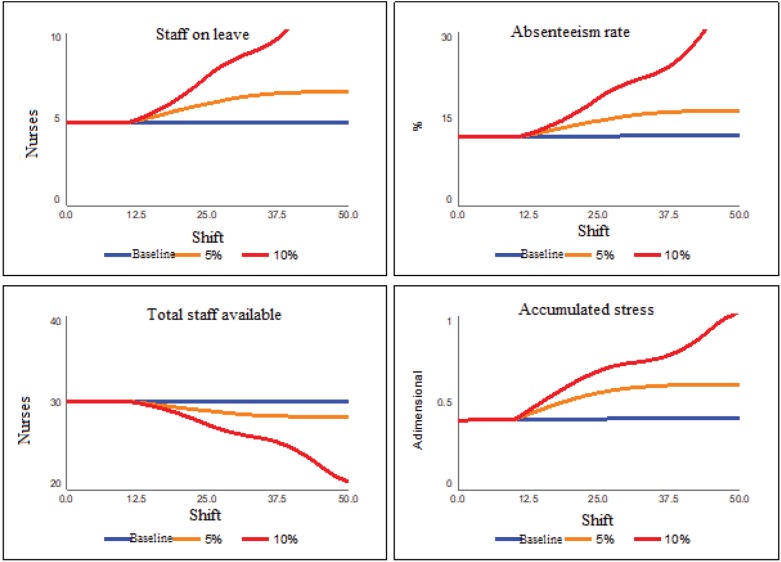
Increased team scenarios

The next set of scenarios tries to present the impact of the increase of one more nurse to the shift team, from the 10th shift, again considering the demand increases of 5% and 10%. Figure 4 presents the results of the second set of scenarios.

With a policy of increasing teams of 27 individuals who attend shifts, which should be composed of 30 to 36 professionals, the teams will have one more component and the service will be milder. With a composition of 36 effective professionals, nurses and technicians will not have to cover absences or leaves due to absenteeism. In this way, the time spent in care will be better divided, thus avoiding the physical and emotional overload of the workers involved. Thus, according to Figure 4, even with increases in the rate of care performed, the increase of the team leads to a considerable reduction of stress, practically dissipating it completely in the 50-shift period, in the case of a 5% increase. In parallel, the absenteeism index - for this same scenario of 5% - reduces to a value close to the average of the sector, approximately 5%.

**Figure 5:**
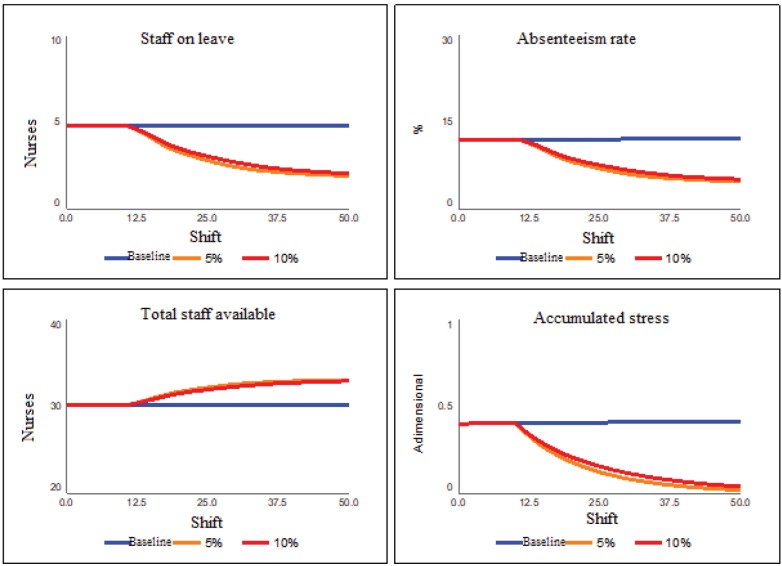
Scenarios of the policy of reduction of hours of service per bed

Lastly, the last set of scenarios tests the impacts of a policy of reduction of hours of care performed per bed, relative to the measurement of absenteeism in the unit, maintaining the size of the team in five nurses. In other words, from the follow-up of the absenteeism index, the number of hours of service per bed is reduced by 20% (that is, from 11 hours per bed per shift to 8.8 hours per bed per shift). Figure 5 presents the results of this scenario for the same 5% and 10% increases in workload.

As can be seen in Figure 5, even with considerable increases in the demand (10%), the absenteeism rate stabilizes at approximately 5%. The staff on leave also stabilizes in two people per shift, as well as the accumulated stress is almost completely dissipated. This scenario presents limitations, since the service provided depends on the evolution of each case. To the extent that the professional manages the time spent in the consultations, the better will be his/her efficiency to face the workload.

## Discussion

This study presents the dynamics of stress generation, accumulation and reduction in the nursing team of an oncology sector and the absenteeism using a system dynamics model. The model used information and insights resulting from dialogues with some professionals in this sector.

It consisted of multiple feedback cycles containing a diverse range of components, including the number of team members, the effect of work leaves, the effect of stress on teams, the workload, the patient care and the rest hours.

The model offers data that allows the manager to control the demands of the work and to follow the evolution of stress among these professionals. In addition, it is possible to predict trends for future scenarios under various scenarios^(^ ^14^ ^)^, such as the situation of US military personnel with post-traumatic stress disorder and a possible aggravation of this situation due to the country's involvement in new conflicts.

One of the key values of systems models is that they show how a set of changes affects many aspects of a complex structure^(^ ^13^ ^)^. The creation of mathematical models represents the productive chain and favors to diagnose and to monitor problematic points in its structure using computational simulations, employing an easy-to-understand representation.

A unique perspective is offered to researchers and practitioners to view stress in the workplace as a dynamic process^(^ ^7^ ^)^. Multiple cycles of recursive feedback are made available for orientation and development of policies and programs within complex organizational contexts.

The demands presented in the model portray real situations of professionals that coexist with stressors and address issues of daily impact and organizational conditions. Improvement of physical and mental conditions and reduction of stress can be achieved by encouraging positive relationships in the workplace, motivation of employees through appropriate working conditions, positive motivation by the bosses, and objective evaluation of work performance^(^ ^15^ ^)^.

Lack of interest in addressing stress at work by both the employer and the employee can compromise the health and performance of the professional, which can impact on a variety of physiological, psychological and behavioral consequences. Organizations can focus on building good levels of employee performance if these effects are addressed, thus promoting the development of a better society as a whole^(^ ^16^ ^)^.

It is up to the organizations to adopt management policies aimed at improving the conditions of the health sector^(^ ^17^ ^)^, because at the moment they conclude that it was in the work that the suffering and the wear and tear were generated, the subject deserves attention of the management devices. Meetings with teams are important for planning activities that seek the valuation of different knowledge with emphasis on the experiences of professionals in order to maintain the workers’ health.

Identifying and monitoring workplace problems using systems dynamics models can provide greater safety to the manager and thereby better monitoring of team work. In addition, a person working in a better work environment is more likely not to be stressed and not to be absent from work compared to a person working in a physically exhausting and psychologically depressing environment^(^ ^18^ ^)^.

Health and wellness management should begin with changing attitudes; the promotion of basic psychological satisfaction (i.e., autonomy, relationship and competence), well-being and health can avoid negative consequences for employees and organizations and can maximize organizational performance^(^ ^19^ ^)^. In addition, interpersonal relationships, dialogue^(^ ^20^ ^)^, and the meeting of the physical and emotional demands of the professionals are factors considered important to improve the quality of life at work.

Another important factor is the fact that patient satisfaction^(^ ^5^ ^)^ is linked to staff absenteeism and, to increase patient satisfaction, managers need to find a way to reduce staff absenteeism in order to avoid burnout and improve the atmosphere in the workplace.

The model, with its dynamics, proposes changes to improve team support, psychosocial aspects, mutual collaboration in the execution of tasks, and work and family balance. Actions that seek to mediate relationship problems in teams are important^(^ ^21^ ^)^, because stress, disagreement and horizontal violence are common in workplaces.

In addition, oncological treatment involves work overload, lack of equipment, long and aggressive treatments, side effects, feelings of despair and panic of patients and death^(^ ^22^ ^)^, thus demanding more involvement, knowledge and emotional balance.

In a long-term perspective, it will be interesting to further explore the issues related to human interrelated factors to better understand the complexities of human behavior for different stress situations. The application of the model in other environments and a sample with a larger “n” of participants could enrich the quality of the model.

The adoption of a model with more elements and greater relations increases the validation in a significant way. The scientific literature on management and psychology does not always agree on how and to what extent variables influence each other, and longitudinal empirical studies are still lacking. Despite the barriers mentioned, there is a sign that the development of computer simulation tools offers considerable potential for the management of health services.

Specially, the results of this study may contribute to nursing science in designing reform initiatives related to staff management, work overload, stress management among nurses, and to reduce the negative consequences of stress in the quality of life at work, in the provision of service and in the costs.

## Conclusion

The presented system dynamics model is able to represent the complex mechanisms of feedback involving the mental processes of workers at an oncology nursing unit. The dynamic representation of stress generation, accumulation and reduction, as well as the effect on absenteeism in the nursing team meets the required requirements and the relation of stocks and flows responds in a coherent way to the changes proposed in the simulations.

The model provides the manager with a tool to dynamically monitor the demands of the sector, presenting the current situation and simulating future situations, such as the effects on staff reduction and hiring, control of slack, the distribution of tasks in an egalitarian way and the variations in stress levels according to their decisions.

Given the complexity of the nursing service and despite the limitations, it is expected that the present study will provide another alternative for nursing managers in coping with stress, absenteeism, and improving the quality of life at work.

Finally, additional research should be encouraged by applying the systems dynamics methodology to study stress and consequences in the work environment.
